# Supplementary figures and images for: In vivo identification and validation of novel potential predictors for human cardiovascular diseases
Source: PLoS One. 2021 Dec 17;16(12):e0261572. doi: 10.1371/journal.pone.0261572 (PMC8682894; doi:10.1371/journal.pone.0261572)

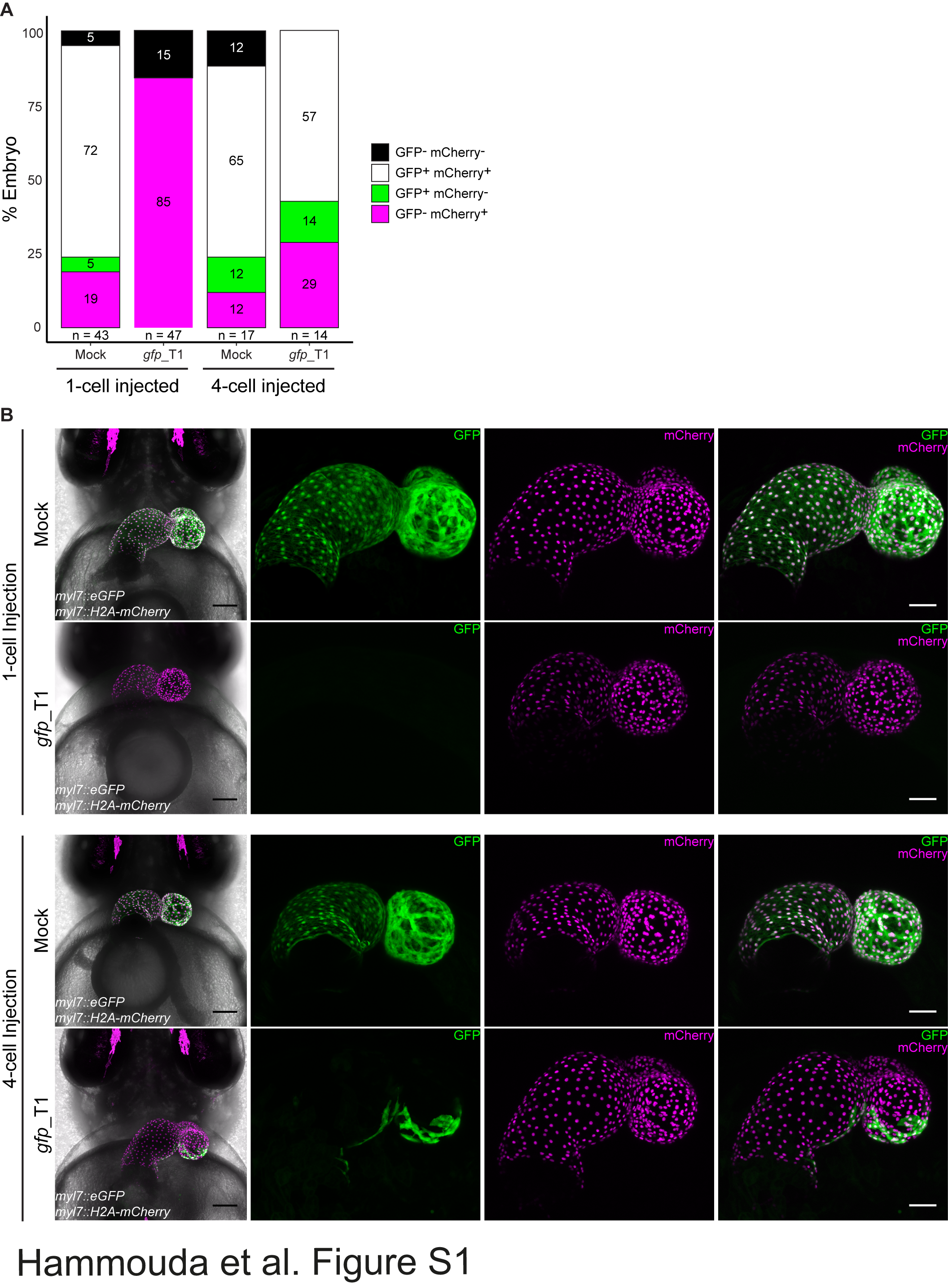

Supplement: S1 Fig — (A) Distribution of reporter expression in mock injected and gfp_T1 crispants of the myl7::eGFP myl7::H2A-mCherry reporter line (4 dpf). Embryos were injected either at the 1-cell or 4-cell stage. Note: complete lack of GFP-expressing embryos when injected at the 1-cell stage. Biological replicates for each group is denoted (n). (B) Confocal images (mirrored) of GFP expression in mock-injected and gfp crispant embryo hearts of the myl7::eGFP myl7::H2A-mCherry reporter line (7 dpf). Embryos were injected either at the 1-cell or 4-cell stage. Note: complete loss of GFP expression when injected at the 1-cell stage (n = 8/8), while mosaic expression when injected at 4-cell stage (n = 4/4). Scale bars: 100 μm (First panel on left) and 50 μm (blow-up images). (TIF) [file pone.0261572.s001.tif]

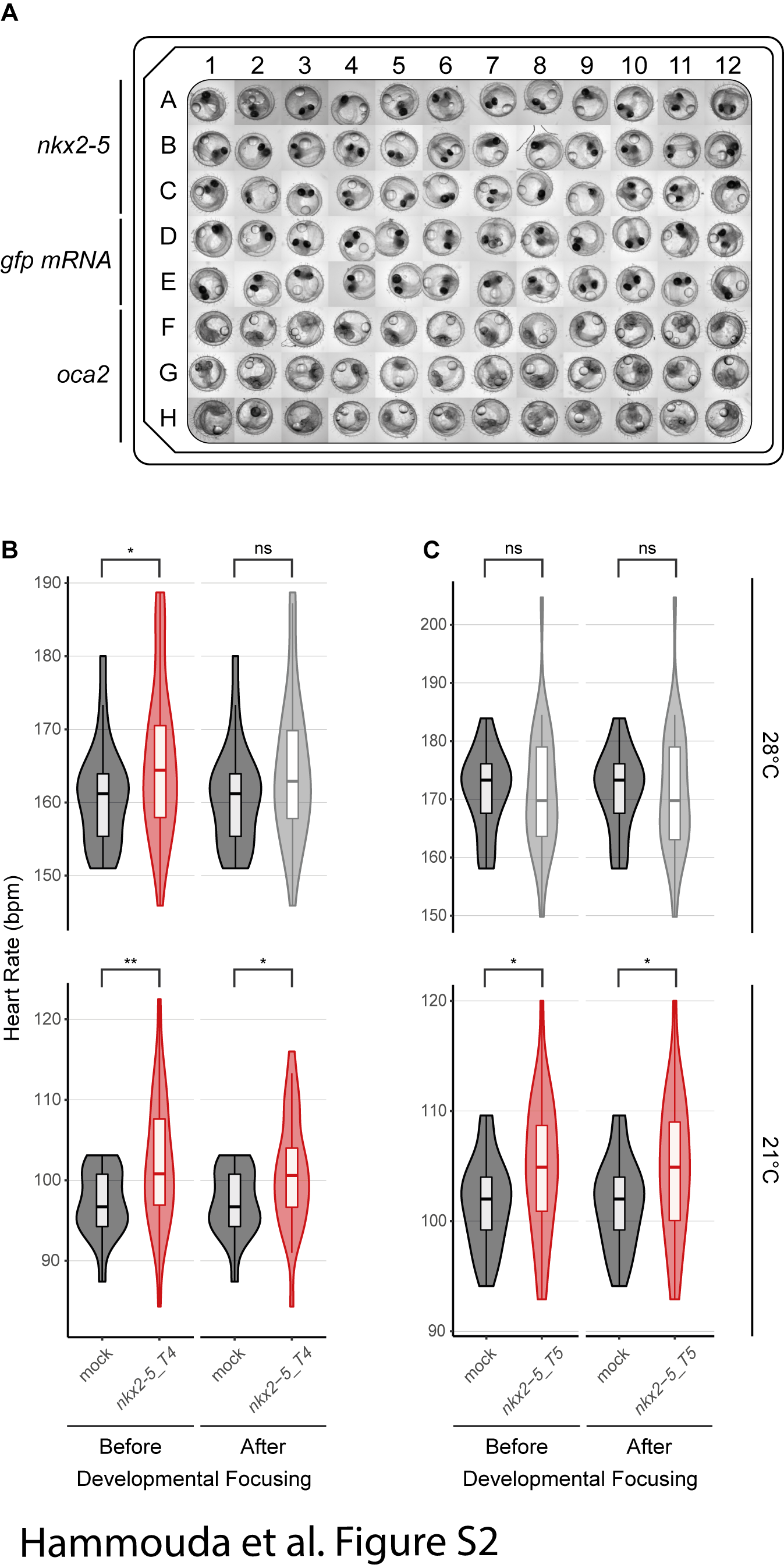

Supplement: S2 Fig — (A) Overview of 96-well plate with embryos (4 dpf) injected with sgRNA against nkx2-5 or oca2, as well as embryos mock injected with GFP mRNA (Fig 1D). Note the loss of eye pigmentation in oca2 crispant embryos. (B-C) Heart rate measurements of GFP-injected (Mock; dark grey) and nkx2-5 crispant embryos (4 dpf) ((B) second replicate of nxk2-5_T4; (C) different sgRNA nkx2-5_T5 targeting same region of interest) at 21 and 28°C, before and after exclusion of severely affected embryos (< stage 28; developmental focusing). Significant differences are shown in red and were determined by two-tailed Student’s t-test; *p < 0.05, **p < 0.01, ns (not significant; light grey). For biological replicates see Source Data S2 Fig in S1 Data. (TIF) [file pone.0261572.s002.tif]

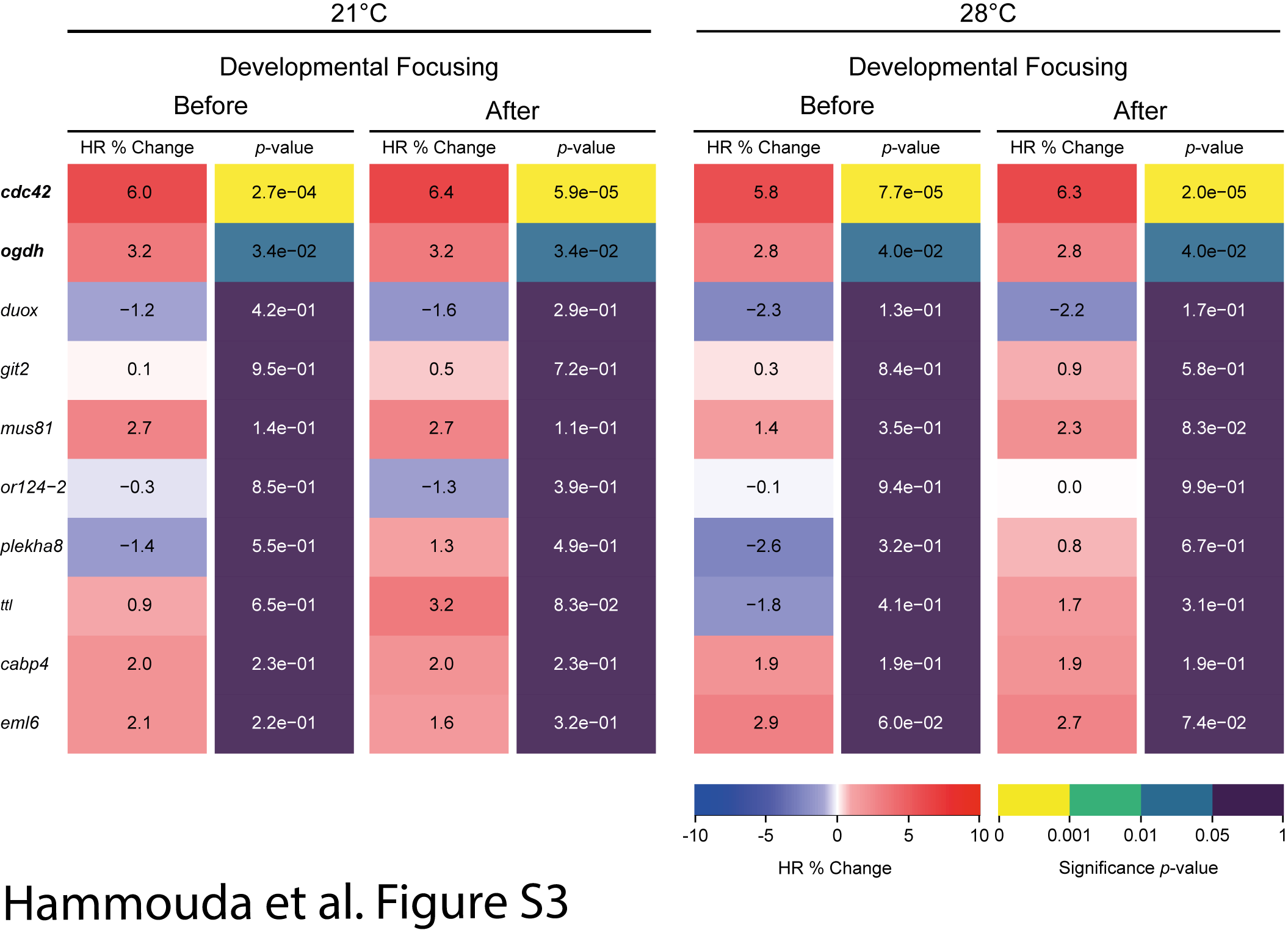

Supplement: S3 Fig — Heatmap quantitative representation of the comparative heart rate analysis between each crispant embryo group and its corresponding control sibling group before and after developmental focusing; for each measured temperature, the percent change in mean heart rate (HR % Change) between crispants and their corresponding control sibling, flanked by the statistical significance (p-value) of the observed change calculated by two-tailed Student’s t-test on the full distribution. Genes showing significant heart rate phenotypes are indicated in bold. For biological replicates see Source Data S3 Fig in S1 Data. (TIF) [file pone.0261572.s003.tif]

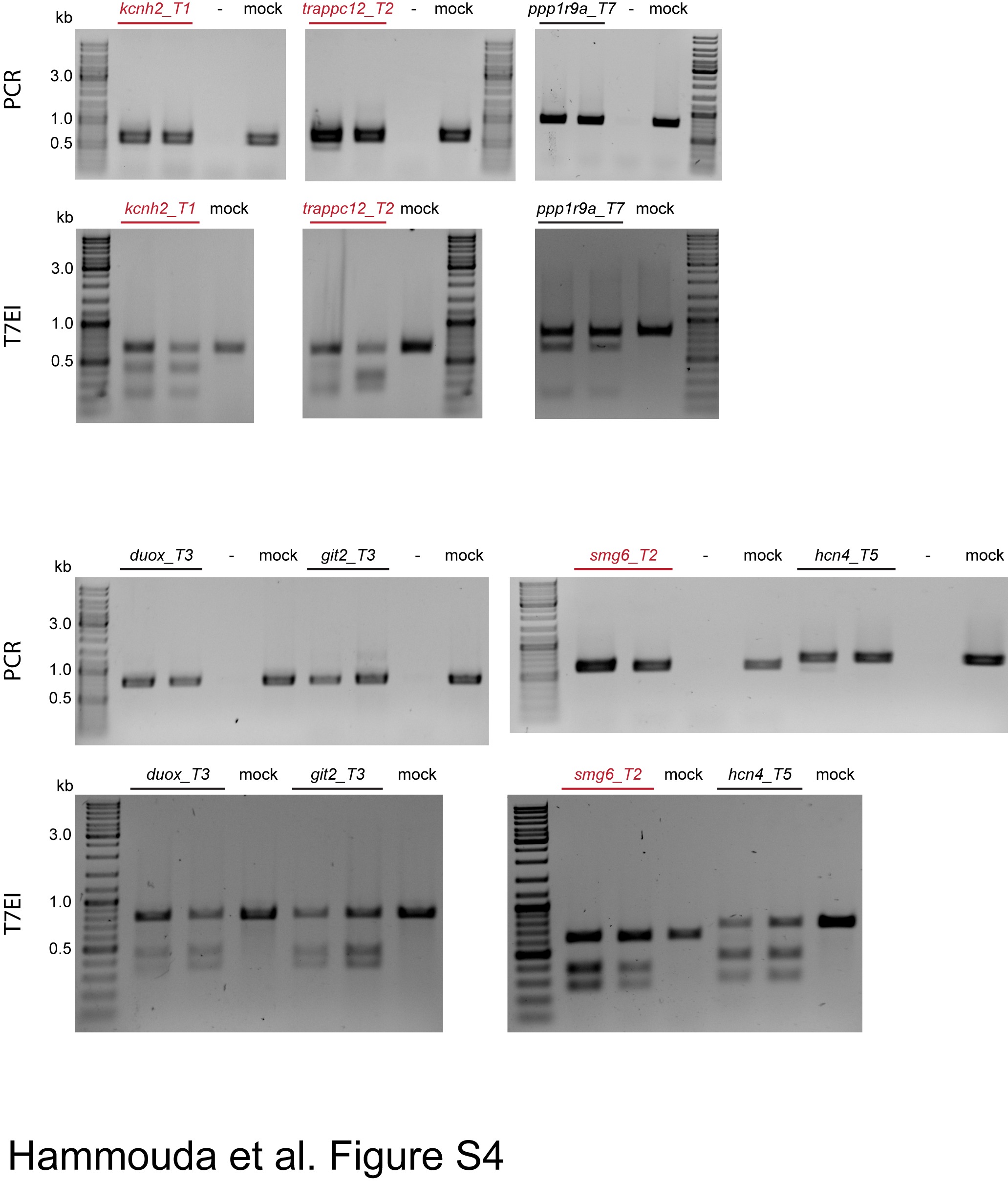

Supplement: S4 Fig — Representative examples of validated CRISPR-mediated gene targeting in vivo, confirming successful heiCas9 targeting and cleavage via sgRNAs employed. PCR amplification of target locus followed by T7EI mismatch cleavage assay (T7EI) demonstrates successful in vivo gene editing of target genes yielding a heart rate phenotype (red) as well as genes not yielding a heart rate phenotype (black). Per sgRNA, two randomly selected individual embryos were genotyped, while including a negative control (water; -) as well as a mock-injection control with GFP mRNA only (mock). (TIF) [file pone.0261572.s004.tif]

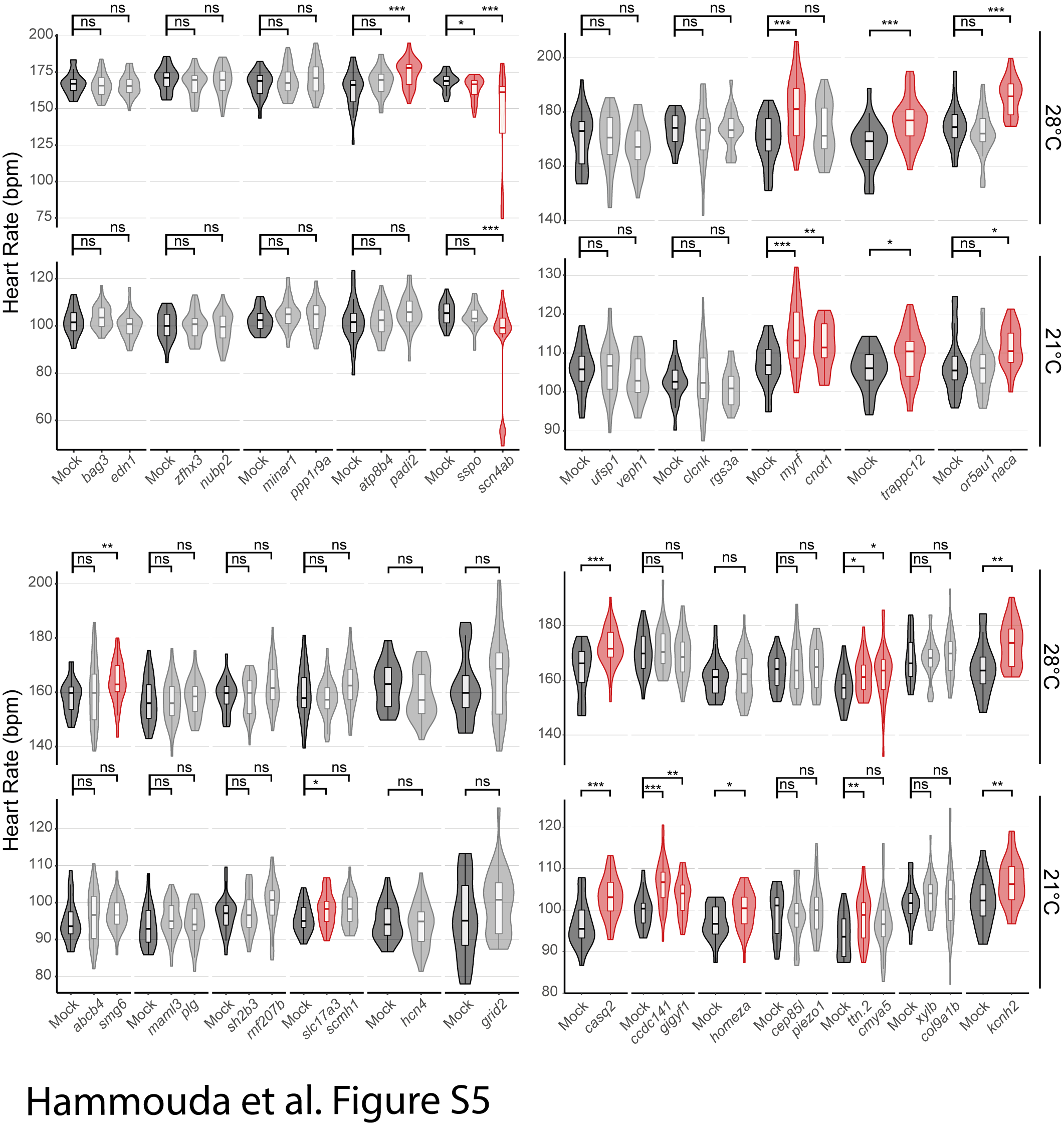

Supplement: S5 Fig — Heart rate measurements (beats per minute, bpm) of GFP-injected (Mock; dark grey) and corresponding sibling crispant embryos (4 dpf) at 21 and 28°C after developmental focusing (also see heatmap representation of the data in Fig 3A). Different experimental plates are represented by breaks on the x-axis. Significant differences in mean heart rates were determined between each crispant embryo group and its corresponding sibling control group by two-tailed Student’s t-test; *p < 0.05, **p < 0.01, ***p < 0.001, ns (not significant). Red groups correspond to crispants showing significant heart rate phenotypes, and light grey groups correspond to crispants showing no significant heart rate phenotype. For biological replicates see Source Data S5 Fig in S1 Data. (TIF) [file pone.0261572.s005.tif]

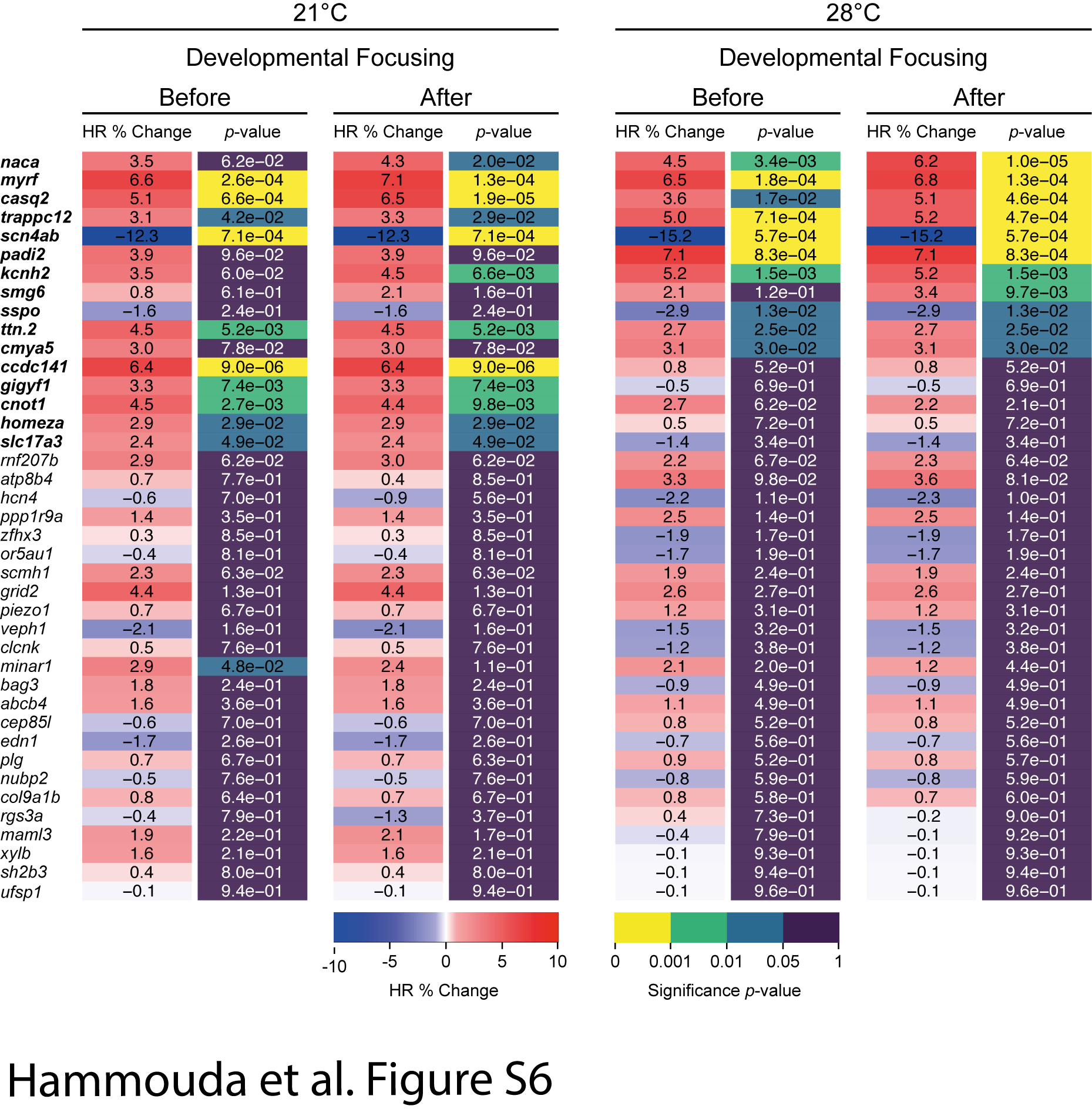

Supplement: S6 Fig — Heatmap quantitative representation of the comparative heart rate analysis between each crispant embryo group and its corresponding control sibling group before and after developmental focusing; for each measured temperature, the percent change in mean heart rate (HR % Change) between crispants and their corresponding control siblings, flanked by the statistical significance (p-value) of the observed change, calculated by two-tailed Student’s t-test on the full distribution. Genes showing significantly different heart rate phenotypes are indicated in bold. For biological replicates see Source Data S5 Fig in S1 Data. (TIF) [file pone.0261572.s006.tif]
