# Supplementary material for: In vivo identification and validation of novel potential predictors for human cardiovascular diseases
Source: PLoS One. 2021 Dec 17;16(12):e0261572. doi: 10.1371/journal.pone.0261572 (PMC8682894; doi:10.1371/journal.pone.0261572)
Supplement: S1 Table — (DOCX) [file pone.0261572.s009.docx]

## S1 Table. List of sgRNAs used.

| **sgRNA** | **Target sequence [PAM]** |
| --- | --- |
| *abcb4_T7* | ATGTCCCTGAGTGTGAAGAG[TGG] |
| *atp8b4_T2* | TAAGTTCTACGATAACACCC[TGG] |
| *bag3_T1* | GAGCAGGGCGCCGTTCACCC[GGG] |
| *cabp4_T9* | GAATTTGACTACGATGCAGA[TGG] |
| *casq2_T1* | TGTTGTCTGGGATGGGTTCG[TGG] |
| *ccdc141_T1* | CCAGGAACCTGAAGGTTGTC[AGG] |
| *cdc42_T2* | AGAGCGGAGAGAAACTGGCT[CGG] |
| *cep85l _T5* | AACCCAGGACTTCTCAGATA[GGG] |
| *clcnk_T10* | TTCTCCACTGGAGTAGTTTT[TGG] |
| *cmya5_T1* | GTAGAACAGGTAATTCTCGT[TGG] |
| *cnot1_T5* | GCCAAGTGTTGACAATACTG[AGG] |
| *col9a1b_T2* | GACGGACGCGTAGGCATTCC[AGG] |
| *duox_T3* | CATTCGGCACGCTTTCTCTA[AGG] |
| *edn1_T4* | GCCGACGGAGTCTGCGCGGA[GGG] |
| *eml6_T2* | CTGCGCTGTTCGCACGCTAA[AGG] |
| *gfp_T1* | AGCACTGCACGCCGTAGGTC[AGG] |
| *gigyf1_T3* | GAATGAACCGGCATGAACGC[CGG] |
| *git2_T3* | TAAACGCCTTCGAAACACGG[AGG] |
| *grid2_T8* | AAAGGCTACGGCTAAGGGTC[TGG] |
| *hcn4_T5* | AAACTCCCTTCGAACTTGTG[AGG] |
| *homeza_T1* | GCTACCAGCAGGTGCGAGAT[TGG] |
| *kcnh2_T1* | CATCACTGCTGGGAGAACCG[GGG] |
| *maml3_T19* | TCATGTAAGGTGTCATCATA[GGG] |
| *minar1_T1* | GTTGCCGTCGGCGACGCGTA[GGG] |
| *mus81_T7* | AAGAGGATGGACGACCTCTG[TGG] |
| *myrf_T1* | CCTTATTGGAGTCCATATTG[TGG] |
| *naca_T1* | TTGGTCTTAGGCAAGTAACG[GGG] |
| *nkx2-5_T4* | GCCGCGGGTCCTCTTCTCCC[AGG] |
| *nkx2-5_T5* | CGGACAGACCCAAGCCCCGG[AGG] |
| *nubp2_T4* | GAACTGAATGTGGCACTGTT[AGG] |
| *oca2_T3* | TTGCAGGAATCATTCTGTGT[GGG] |
| *ogdh_T4* | CAAAGCTGGACTTGGCCTCA[GGG] |
| *or124-2_T1* | GCCCACGTGGTCTCGTACGC[CGG] |
| *or5au1_T2* | GTCATGGACTCTTGCCTGTA[TGG] |
| *padi2_T4* | AGACTGGTATTACGGTTAAG[AGG] |
| *piezo1_T1* | TTGGGAGCCCGGATGTAGTT[AGG] |
| *plekha8_T9* | GCGGTGAAGCTTTCATAACA[CGG] |
| *plg_T1* | GGCAACGGGGCCAATTATCG[AGG] |
| *ppp1r9a_T7* | TGGTTCTTAGGAGATTCGGG[TGG] |
| *rgs3a_T6* | GATCAAGTCACAGTCCAAGA[TGG] |
| *rnf207b_T2* | TAGACTTGTTTGTACTGATC[TGG] |
| *scmh1_T2* | TCTGACCTGCGCTGTACGAG[TGG] |
| *scn4ab_T2* | TTAGGCTTAGCAATCCGCAA[CGG] |
| *sh2b3_T1* | AAGAAGTCCGTCGCTGCAAT[CGG] |
| *slc17a3_T5* | GTCTTGGCGCCGATTGTGAC[AGG] |
| *smg6_T2* | AAAGATAAAACCAAGGGCGT[AGG] |
| *sspo_T4* | GTGCACCAAGTCATGTGGTT[GGG] |
| *trappc12_T2* | CAACACGCAGTGCCTCAAGC[TGG] |
| *ttl_T1* | GCTGGTAAATTACTACAGAG[GGG] |
| *ttn.2_T1* | GAGCTAGCTGTCAAAGCCAT[GGG] |
| *ufsp1_T1* | GCTGGAGAGCATCGCAGTCC[AGG] |
| *veph1_T1* | GGCTCTGGTGGAGGTGTCCC[AGG] |
| *xylb_T1* | TTTCCAGTGTCAGTACCTAC[AGG] |
| *zfhx3_T1* | AGCTGAGCGCACCCTGCCTG[AGG] |

sgRNA target sites given in 5’-3’ direction, protospacer adjacent motif (PAM) in square brackets.
