# Supplementary material for: In vivo identification and validation of novel potential predictors for human cardiovascular diseases
Source: PLoS One. 2021 Dec 17;16(12):e0261572. doi: 10.1371/journal.pone.0261572 (PMC8682894; doi:10.1371/journal.pone.0261572)
Supplement: S2 Table — (DOCX) [file pone.0261572.s010.docx]

## S2 Table. List of primers used for genotyping by PCR.

| **Target Gene** | **Primers (5’ – 3’ direction)** |
| --- | --- |
| *abcb4* *Lf* | CCTCTGCAGAAGCTGGATGG |
| *abcb4* *Lr* | TTTGCGATGGCGTGTGAGCG |
| *atp8b4* *Lf* | ACCTACAGTCAAAGGGAAATGAGC |
| *atp8b4* *Lr* | ACGCTGCCTCGTCTGGTGAC |
| *bag3* *Lf* | CTTCGCTTAGGAGCCAGTCC |
| *bag3* *Lr* | CAGTATGGTCTGAACCCGCC |
| *cabp4* *Lf* | GTGCCATCTTGCCATGCTGC |
| *cabp4* *Lr* | ATCCTGCTGCTCCTGGTCCC |
| *casq2* *Lf* | CGAACAGGTCAAACCGTGTG |
| *casq2* *Lr* | GGAACTTGCACAAACGGACC |
| *ccdc141 Lf* | GGCTACGGATGAAGGAGCTC |
| *ccdc141 Lr* | TCCTGGCTAAGTGAAGCAGC |
| *cdc42 Lf* | TCCAGAGCTGCGAGGATGGC |
| *cdc42 Lr* | CCAGCCGCTCTGTTCTGGGC |
| *cep85l Lf* | AGGAGTGAGCTGAGGATGGG |
| *cep85l Lr* | GCATCCTCACTCTAGCCGGG |
| *clcnk Lf* | GATTCATGCTGTGTTCCCGC |
| *clcnk Lr* | CCACTGAGTTGGTCGCTTTG |
| *cmya5 Lf* | TTTGTGGGAGCTCGTCTGGG |
| *cmya5 Lr* | GCCACGCTGAGATGGAGCCC |
| *cnot1 Lf* | CCGGTTGTGGCGCCTTGAGG |
| *cnot1 Lr* | GCTTTGGGTCTGGCACTGCG |
| *col9a1b Lf* | AGGGACTCACTTACCGGAAGCCC |
| *col9a1b Lr* | CTGGTCCTCAGGGAGTGGCAGG |
| *duox Lf* | GACGCTGTCAGCTCGCACTG |
| *duox Lr* | GGTCCACTCATCGCTCCCTC |
| *edn1 Lf* | TCTCCGTGCTGTCAGTGTTC |
| *edn1 Lr* | ATGCTGGTTGCCATGGAGTC |
| *eml6 Lf* | ACGCCACGGACAGCATGGTC |
| *eml6 Lr* | TCACTGACAGCTTTGAAGGTTGACG |
| *gfp Lf* | GTGAGCAAGGGCGAGGAGCT |
| *gfp Lr* | CTTGTACAGCTCGTCCATGC |
| *gigyf1 Lf* | ACAGGAGATAGCAGCAGCAG |
| *gigyf1 Lr* | CGTCCCTGATTGTAGATTTGCG |
| *git2 Lf* | TTGCTGTCGCCGCTGCTCTG |
| *git2 Lr* | GGGCTCATGGGACCTTATGTTGG |
| *grid2 Lf* | ACCTACAGACCTGTAACTCCTTCGC |
| *grid2 Lr* | ACCTGGAGCACCTACACCACAGG |
| *hcn4 Lf* | GACCCGTCAAGTGCCACAGG |
| *hcn4 Lr* | CACACCCTCCTTGTCCCTTG |
| *homeza Lf* | GCCAAGGAAGACCAAAGAGC |
| *homeza Lr* | TCACAAACCTCGCTCTAGGC |
| *kcnh2 Lf* | TCGCCCAGGCAAGTCCAACG |
| *kcnh2 Lr* | ACCTCATCGCCACTTGAGTGTG |
| *maml3 Lf* | CGGCAGCCATGTTGTCGTAC |
| *maml3 Lr* | AAGCGGGTTATGTGTGCTGC |
| *minar1 Lf* | CGGTCAGCATGAACTAAGCTTCGG |
| *minar1 Lr* | TGGAGACGGACAGTGATTCCAGTGG |
| *mus81 Lf* | GAAGACGACGAGACGGCCGG |
| *mus81 Lr* | TGACCCGAACAGTACCTTTGTG |
| *myrf Lf* | GGTCCACCAACCGCCTGCC |
| *myrf Lr* | AGCATCCTAGCATTGCAGCC |
| *naca Lf* | TCGCTCTCCTCCTGTACTGTAGGG |
| *naca Lr* | TCTGAGTCTACTGAAGCCAGCC |
| *nkx2-5 lf* | TAGCTTTGAGGCCTGCAGAC |
| *nkx2-5 lr* | CGCATAGTTCGTGTTGCAGG |
| *nubp2 Lf* | ACTGATGAGCCAGCCAACCC |
| *nubp2 Lr* | TGGTCTGTGGCCACATGGTG |
| *oca2 Lf* | GTTAAAACAGTTTCTTAAAAAGAACAGGA |
| *oca2 Lr* | AGCAGAAGAAATGACTCAACATTTTG |
| *ogdh Lf* | GAACACTCGTCTGGCCGGCC |
| *ogdh Lr* | TACAGCAAGTCCCGCCCACG |
| *or124-2 Lf* | TGAGGCTCAGACCCTCCTGG |
| *or124-2 Lr* | ATGCGGTCCGGATGAGCTGG |
| *or5au1 Lf* | GCATTCAGCAGTTCTTCTGTCTTC |
| *or5au1 Lr* | GGCTTTGATAGATCTGTGTGGAAGC |
| *padi2 Lf* | GCATGACACTACCTGAGAATGAAAC |
| *padi2 Lr* | AGCCTCAGTCATCTCAGTAAAGC |
| *piezo1 Lf* | GCAGCAGCAGGAAGGTCAGG |
| *piezo1 Lr* | CCGCTGCATTAGACCACTGC |
| *plekha8 Lf* | CTGGCACCTTCCTGTCCAGC |
| *plekha8 Lr* | ACGGAAGTCGCCCAAGTCCG |
| *plg Lf* | CACAAACACAGCCGCACGCC |
| *plg Lr* | GTGGGCGTGTCCGAGTCGAC |
| *ppp1r9a Lf* | CGAGGTCCAACCGAGGCAGC |
| *ppp1r9a Lr* | TCTCTGTCAGATCTGAGCCGGG |
| *rgs3a Lf* | TGAGCTGTGTTCGCCCACTC |
| *rgs3a Lr* | GGAGGGCTTCAACTGTGAGG |
| *rnf207b Lf* | GCAGAAGCCTCCCATTGACG |
| *rnf207b Lr* | CCTCTGGCTGACCGCTCCTG |
| *scmh1 Lf* | ACTCTGTTCGAGCGCCTCCC |
| *scmh1 Lr* | AGGCGCATCCTGCAGCTTCC |
| *scn4ab Lf* | TCACCACTTCTGCAGGATGG |
| *scn4ab Lr* | GAGGCGTAGTGATCTGACGG |
| *sh2b3 Lf* | TTTCTGCCGGCATCTGCTCC |
| *sh2b3 Lr* | CCTGCTGGTCGTTGTCCGTC |
| *slc17a3 Lf* | CCGCTGTGCCCAGGAGCAAG |
| *slc17a3 Lr* | AGGAAACGACCCGTGACCAC |
| *smg6 Lf* | CCCAGCATCAGTGGAGGTGC |
| *smg6 Lr* | CCCTGAGCCTCAGTCCCAGC |
| *sspo Lf* | TGCAGTCGAGGGTCAGTGGTCG |
| *sspo Lr* | CACAGGATCGCGGGCAAGAAGG |
| *trappc12 Lf* | GTGCCTGCTGTACCTGGGCC |
| *trappc12 Lr* | ACCACCACCCTCTCTCCCAGC |
| *ttl Lf* | CCGGGCGGGACCAACAACTG |
| *ttl Lr* | GGAGTCAGACAGCTCTGGGC |
| *ttn.2 Lf* | CAGAGCTGGACGGACATCGG |
| *ttn.2 Lr* | GGAGCGCTGACGTCTGCCTC |
| *ufsp1 Lf* | TGGGAAAGGACTAGAGGAGGG |
| *ufsp1 Lr* | GACACTCGCTTCCAGGACAC |
| *veph1 Lf* | TCTGTCAGAGCGGATGCAAG |
| *veph1 Lr* | AGTCTCTGATCGGGATGCAAC |
| *xylb Lf* | CCTGCAGCAACATGGCAGTGTG |
| *xylb Lr* | ACCATCACTGTGGTCAATGGCTGC |
| *zfhx3 Lf* | GGCTGGGTGGTGGGTGTCAG |
| *zfhx3 Lr* | TGTGCGGCTAGGTGGTGGAC |
