# Supplementary material for: In vivo identification and validation of novel potential predictors for human cardiovascular diseases
Source: PLoS One. 2021 Dec 17;16(12):e0261572. doi: 10.1371/journal.pone.0261572 (PMC8682894; doi:10.1371/journal.pone.0261572)
Supplement: S1 Data — (DOCX) [file pone.0261572.s011.docx]

**Source Data Fig 1D. Biological replicates for Fig 1D.**

| **Gene_sgRNA** | **Ns before DF** | | **Ns after DF** | | **Ns mock** | |
| --- | --- | --- | --- | --- | --- | --- |
|  | **21C** | **28C** | **21C** | **28C** | **21C** | **28C** |
| *nkx2-5_T4* | 34 | 34 | 31 | 31 | 22 | 23 |
| *oca2_T1* | 35 | 35 | 35 | 35 |  |  |

Ns correspond to number of scored embryos per condition. before DF = before developmental focusing (i.e. all scored embryos). after DF = after developmental focusing (i.e. only scored embryos looking older than stage 28).

**Source Data Fig S2. Biological replicates for Fig S2 B-C.**

| **Gene_sgRNA** | **Ns before DF** | | **Ns after DF** | | **Ns mock** | |
| --- | --- | --- | --- | --- | --- | --- |
|  | **21C** | **28C** | **21C** | **28C** | **21C** | **28C** |
| *nkx2-5_T4* | 34 | 36 | 31 | 33 | 19 | 23 |
| *nkx2-5_T5* | 33 | 33 | 31 | 31 | 22 | 21 |

Ns correspond to number of scored embryos per condition. before DF = before developmental focusing (i.e. all scored embryos). after DF = after developmental focusing (i.e. only scored embryos looking older than stage 28).

**Source Data Fig S3.** **Biological replicates for Fig 2 and S3.**

| **Gene** | **Ns before DF** | | **Ns after DF** | | **Ns mock before -> after DF** | |
| --- | --- | --- | --- | --- | --- | --- |
|  | **21C** | **28C** | **21C** | **28C** | **21C** | **28C** |
| *cdc42* | 32 | 32 | 29 | 30 | 22 | 20 |
| *ogdh* | 31 | 33 | 31 | 33 |  |  |
| *duox* | 32 | 33 | 27 | 28 | 19 | 21 |
| *git2* | 35 | 33 | 33 | 31 |  |  |
| *mus81* | 27 | 33 | 25 | 31 | 20 -> 18 | 21 -> 20 |
| *or124-2* | 34 | 34 | 30 | 30 |  |  |
| *plekha8* | 35 | 31 | 29 | 26 | 21 | 23 |
| *ttl* | 33 | 30 | 28 | 24 |  |  |
| *cabp4* | 34 | 34 | 34 | 34 | 21 | 21 |
| *eml6* | 36 | 34 | 34 | 32 |  |  |

Ns correspond to number of scored embryos per condition. before DF = before developmental focusing (i.e. all scored embryos). after DF = after developmental focusing (i.e. only scored embryos looking older than stage 28).

**Source Data Fig S5.** **Biological replicates for Fig 3A, S5 and S6.**

| **Gene** | **Ns before DF** | | **Ns after DF** | | **Ns mock before -> after DF** | |
| --- | --- | --- | --- | --- | --- | --- |
|  | **21C** | **28C** | **21C** | **28C** | **21C** | **28C** |
| *bag3* | 32 | 33 | 32 | 33 | 22 | 24 |
| *edn1* | 35 | 34 | 35 | 34 |  |  |
| *zfhx3* | 33 | 35 | 33 | 35 | 23 | 22 |
| *nubp2* | 36 | 34 | 36 | 34 |  |  |
| *minar1* | 33 | 33 | 30 | 30 | 24 | 22 |
| *ppp1r9a* | 32 | 34 | 32 | 34 |  |  |
| *atp8b4* | 35 | 34 | 32 | 31 | 24 | 23 |
| *padi2* | 31 | 36 | 31 | 36 |  |  |
| *sspo* | 33 | 32 | 33 | 32 | 22 | 22 |
| *scn4ab* | 35 | 32 | 35 | 32 |  |  |
| *ufsp1* | 33 | 32 | 33 | 32 | 23 | 24 |
| *veph1* | 34 | 35 | 34 | 35 |  |  |
| *clcnk* | 34 | 31 | 34 | 31 | 22 | 23 |
| *rgs3a* | 26 | 28 | 21 | 23 |  |  |
| *myrf* | 34 | 34 | 33 | 33 | 24 -> 23 | 23 -> 22 |
| *cnot1* | 36 | 35 | 22 | 22 |  |  |
| *trappc12* | 35 | 35 | 34 | 34 | 23 | 24 |
| *or5au1* | 34 | 33 | 34 | 33 | 22 | 22 |
| *naca* | 33 | 32 | 27 | 27 |  |  |
| *abcb4* | 36 | 35 | 36 | 35 | 23 | 22 |
| *smg6* | 36 | 35 | 30 | 29 |  |  |
| *maml3* | 35 | 35 | 34 | 34 | 23 | 24 |
| *plg* | 35 | 36 | 33 | 34 |  |  |
| *sh2b3* | 35 | 36 | 35 | 36 | 24 | 24 |
| *rnf207b* | 35 | 36 | 33 | 34 |  |  |
| *slc17a3* | 36 | 35 | 36 | 35 | 22 | 24 |
| *scmh1* | 33 | 33 | 33 | 33 |  |  |
| *hcn4* | 35 | 36 | 33 | 34 | 23 | 24 |
| *grid2* | 30 | 34 | 30 | 34 | 23 | 20 |
| *casq2* | 59 | 56 | 53 | 50 | 25 -> 24 | 24 -> 23 |
| *ccdc141* | 36 | 34 | 36 | 34 | 22 | 23 |
| *gigyf1* | 34 | 35 | 34 | 35 |  |  |
| *homeza* | 32 | 35 | 32 | 35 | 19 | 23 |
| *cep85l* | 35 | 36 | 35 | 36 | 22 | 24 |
| *piezo1* | 35 | 33 | 35 | 33 |  |  |
| *ttn.2* | 34 | 35 | 34 | 35 | 23 | 24 |
| *cmya5* | 33 | 34 | 33 | 34 |  |  |
| *xylb* | 35 | 33 | 35 | 33 | 23 | 22 |
| *col9a1b* | 35 | 35 | 34 | 34 |  |  |
| *kcnh2* | 34 | 36 | 34 | 36 | 21 -> 20 | 21 |

Ns correspond to number of scored embryos per condition. before DF = before developmental focusing (i.e. all scored embryos). after DF = after developmental focusing (i.e. only scored embryos looking older than stage 28).
