# Supplementary material for: In vivo identification and validation of novel potential predictors for human cardiovascular diseases
Source: PLoS One. 2021 Dec 17;16(12):e0261572. doi: 10.1371/journal.pone.0261572 (PMC8682894; doi:10.1371/journal.pone.0261572)
Supplement: Attachment — Submitted filename: Hammouda_etal_PLOS ONE_Response to Reviewers.docx [file pone.0261572.s012.docx]

**Reviewer #1:**

The article by Hammouda et al. describes functional validation of candidates genes identified for human cardiovascular diseases through GWAS studies in Medaka embryos. Authors layout a plan where they first identified the candidate genes from human GWAS data. Then they identified the appropriate Medaka homolog for the gene and, using CRISPR/Cas9, targeted the gene. Surviving embryos were allowed to grow for four days when they had a stable heartbeat/rate and then imaged in a 96 well format dish with changes in the heart rate compared to the controls. Conceptually it is a nice design of experiments to validate candidate gene function in fish embryos. Still, I am concerned that it should not be a misused approach/technique similar to morpholinos. I have the following concerns:
1: All of the analysis is carried out in F0 animals which are mosaics, and it is hard to predict the cutting/targeting efficiency for each gRNA. Authors should provide additional evidence on cutting efficiencies for their most potent and weakest phenotype carrying animals and rule out the phenotypes/changes in heart rate are not mosaicism based.

*We thank the referee for giving us the opportunity to address the concerns he raised. We are fully aware of those potential problems and have consequently addressed them deeply. We tested the targeting efficiency of each sgRNA employed and analysed editing of the respective loci in injected and control embryos respectively. As suggested by the referee we are now providing these data as new supporting information in the revised version of the manuscript.*

*Injection of Cas9 and short guide RNAs into early embryos has been shown to result in a mosaic action in other model systems. There are two levels of mosaicism to be considered here: Mosaicism of edited and unedited cells on the one hand and mosaicism of edited alleles on the other.*

*In all our experiments we are using a modified Cas9 (HeiTag enhanced Cas9, BioRxiv* ***doi:*** [*https://doi.org/10.1101/2021.05.27.445956*](https://doi.org/10.1101/2021.05.27.445956)*) that we have developed in the lab. With this HeiCas9 we achieve efficient editing already at the 1-cell stage. This results in the uniform editing in the tissue of interest. We control the uniformity of editing of GFP specifically expressed in the heart and show its complete loss in response to the protocol as control for the efficiency of the procedure employed. Mosaicism can only be forced when injecting one out of four cells at a later stage of development.*

*Due to its immediate actions the number of alleles generated (allelic mosaicism) is substantially lower in comparison to other, routinely used, Cas9 variants.*

*Taken together, these data strongly argue for the specificity of the observed effects as consequence of the interference with the tested candidate gene. Those effects were specific for heart-related candidate genes and were never observed in negative control genes or in the randomly selected genes not related to heart function.* *We have taken care to clearly indicate these crucial prerequisites for the entire study in the revised version of the manuscript.*

2: Temperature-dependent changes in the heart rate of CRISPR animals are a bit of an issue. Many GWAS-linked genes need a "modifier" to set the phenotype; mimicking those conditions in fish embryos by modifying temperature is not the best way. Fish embryo development is highly plastic, and it slows down or accelerates based on how cold or warm the growing conditions are. Perhaps authors should consider combinatorial experiments where Crispants for 2 or 3 genes show more severe phenotypes than the single gene alone.

*We thank the referee for this comment and regret that we failed to clearly describe the procedure and the rationale behind it. The different temperatures were applied after the heart had fully developed and heart rate had reached its plateau (Gierten 2020). The different temperatures were only applied for short periods (sixty minutes) during the actual measurement of the heart rate. The temperature change was an acute measure to probe for the heart response in different environmental conditions. Exposing the embryos to different temperatures was aiming at expanding the range of heart phenotype detection by alteration in heart response or by detecting significantly altered “resting heart rates” at different temperatures. We have taken care to clarify this important point in the revised version of the manuscript.*

*The combinatorial targeting of multiple genes is indeed considered in a follow up study.*

3: In Fig 3C (Bar 3), 40% of randomly selected GWAS genes showed HR phenotype. 57% of HR-specific GWAS genes showed heart phenotype. The sample size for this population is almost half compared to total hGWAS genes. This raises the concern about how effective the method is in deciphering correct information.

*We apparently were not successful in clearly making our point. The hGWAS gene set analysed reflects a set of un-validated genes associated to heart phenotypes in human GWAS studies. Upon genome editing, 40% of those genes yielded a heart rate phenotype in medaka, validating their GWAS association. This rate is double as high as when targeting a random selection of genes (20%). The high rate of heart rate effects in the random selection is entirely due to the targeting of heart related genes in this group (that were intentionally not discriminated against). As a consequence, and not surprisingly, the targeting of those resulted in a heart rate phenotype in the random control, while none of the others impacted on the heart rate.*

*When focussing only on “heart rate-related” candidates in the same hGWAS candidate gene set 57% exhibited heart rate phenotypes in medaka validating their association experimentally. In the remainder of the non-heart rate-associated candidates, only 18% exhibited heart rate phenotypes in the validation pipeline. We thank the reviewer for pointing out this potential misunderstanding and we have taken care to clarify these aspects throughout the revised version of the manuscript.*

**Reviewer #2:**

In the submitted manuscript, Hammouda and colleagues outline the use of medaka F0 assays to uncover congenital heart disease genes from GWAS studies and other patient-derived data. The expansion of accessible CRISPR-Cas9-based mutagenesis has rendered such approaches increasingly feasible, yet concerted efforts to establish platforms, protocols, workflows, and coordinated testing of candidate genes are in great need for a variety of diseases. The outlined approach by Hammouda and colleagues is therefore timely and an interesting addition to already existing work.

The manuscript is overall technically sound and well-executed, as common for the authors' lab. The work does address an important aspect of disease gene discovery and definitely warrants publication. However, the manuscript is presented in a "medaka bubble", i.e. not considering work that has been ongoing in the past two decades in mouse as well as in zebrafish. While clearly and undisputedly of interest, the paper phrases the issue of CHD association screening as previously neglected or not addressed, which is misleading. The reviewer doesn't think the authors need to render their own (and exciting) work more novel as it is by starting a narrative that their work is filling a so-far underappreciated gap that is uniquely possible in medaka.
With a little bit of editing, the reviewer has no doubt that the manuscript will be well-received in the model organism community after publication.

* Major Points:
1) From the onset, the authors frame their manuscript without any context to the tremendous amount of technical and experimental work that has already been done in the field of discovering CHD genes. The authors refer to their work as addressing a blind spot (i.e. line 246), which is defining an incorrect narrative.
At the very least, the authors should acknowledge previous work that puts their own work in context, i.e. as complementary approach. Large screens have been performed in mice to correlate found mutants with human CHD data (i.e. 10.1093/hmg/ddq21; 10.1161/CIRCIMAGING.113.000451) and are still ongoing. Further, in zebrafish, extensive experiments have been previously performed to characterize cardiac defects (i.e. 10.1242/dev.099796). Further, the entire approach of using F0 crispants for phenotype readouts, as originally developed in zebrafish, is sidelined and the involved caveats not mentioned (see also below (10.1371/journal.pone.0098186; 10.1101/gr.186379.114; 10.1242/dev.134809 **that defines the term crispants**; or newest developments such as 10.7554/eLife.59683).

*We are happy to widen the focus of our introduction and have taken care to do so and incorporate the suggested aspects and corresponding references in the revised version of the manuscript. We never intended to neglect or sideline their contributions but rather tried to keep the focus. We insist however that only the validation of putative target genes moves them into the spotlight and consequently our approach that is not a screen but rather a validation pipeline represents an important instrument for the inclusion of understudied genes. We have experienced that non-validated candidates have a high tendency of staying understudied. This is illustrated by the fact that the candidate genes from human GWAS are publicly available since 2013, yet many of those were not addressed until now, evident by the lack of characterizations of these genes or experimental reports linking them to heart function. We did not intend in any way to neglect, undermine or ignore previous efforts in gene validations in model organisms and have accordingly taken care to adjust revised version of the manuscript.*

2) The authors chose 10 random genes for targeting as validation (line 135ff). While an interesting approach, the authors then also chose a random exon. That seems like a possibly problematic approach - targeting last exons close to the stop codon could lead to merely hypomorphic or still functional alleles, alternative promoter use could lead to transcripts that don't include mutated exons, etc. The authors should provide further info on guide position (i.e. schematics?) in addition to Table 1 and the used guide sequences.

*We thank the reviewer for pointing this out. We only intended to target the genes at a random location. As also for the hGWAS genes, some polymorphisms that we addressed and targeted were located in various exons, of which could be the last (as for SCN5A/scn4ab). We now included the targeted exon number and the total number of exons for all randomly selected genes in Table 1 of the revised version of the manuscript.*

3) Overall, the authors focus on cardiac rhythm as principal readout, which is an exciting and highly disease-relevant phenotype. However, again, the authors phrase their overall manuscript as aimed at discovering heart disease genes. The authors are encouraged to rephrase their storyline as heart rhythm-focused, which is of high merit in its own regard (and will help bring a more focused audience to the paper).

*We thank the reviewer for pointing this out, as we indeed mainly focus on heart rate phenotypes we adjust our narrative accordingly in the revised manuscript.*

4) What are the mock injections performed as controls? Cas9 will introduce DSB-related stress, so adding a control such as mutating an unrelated (i.e. pigment) gene might be a better control for the experiments than just mock injections (with buffer?).

*We than the referee for this comment since we have indeed been doing both, mock injections as well as control injections mutating a pigment gene. We have taken care to clearly emphasize this in the revised manuscript.*

*Mock injections consist of GFP mRNA (injection tracer) in water. This served as injection control and proven to be sufficient after initial pilot tests shown in our manuscript to minimize unnecessary depletion of heiCas9. It is noteworthy to mention that for every batch of heiCas9 used as well every change in fish generation used for experiments, a negative control as suggested by the referee was performed by targeting a pigment locus (Oca2), and in all attempts (7/7 or even more) no significant heart rate phenotypes were observed, thus ruling out Cas9 or DSB-related stress effect on heart rate.*

5) The authors gauge an AV-block by visual inspection (line 204). This is a highly complex electrophysiological phenotype and issue that requires calcium measurements and imaging/measurement of the cardiac syncytium's conductivity behavior. While reminiscent of an AV-block, the authors are encouraged to tone down their call of this particular phenotype.

*We are happy to follow the suggestion of the referee and have adjusted our claims and description of the phenotype respectively.*

* Minor Points:
1) Introduction: "markers" should possibly rephrased to "causative genes" or the likes.

*We followed the referee’s advice and have changed the term accordingly.*

2) The statement that genes without pre-existing evidence are not being characterized seems once more superficial and not what prior publications show. Suggest rephrasing.

*We have taken care to include any prior publications. In cases where those are absent, our statement seems valid.*

3) That the heart rate of fishes is more in line with the human heart rate (while the entire structure of the heart with two vs four chambers is entirely different and the pacemaker system is only rudimentary similar) seems like a weak argument to promote fishes (in this case particularly medaka) as model compared to mice. Again, the authors don't need to bolster their model - it is valid and interesting as it is.

*We thank the referee for the encouragement and have changed the paragraph accordingly.*

4) cmlc2 should be renamed to the contemporary nomenclature, myl7.

*Done*.

5) Was the targeting of the GFP transgene (line 82) performed on heterozygous, i.e. single-copy, embryos? That should be stated or clarified in relation to the need of biallelic mutagenesis of endogenous loci.

*We took care to clearly state this (heterozygous) in the revised manuscript. The experiment showing efficient bi-allelic targeting is the editing of the pigment locus Oca2 that we present as control.*

6) Throughout the text, the authors are encouraged to add n=x data already into the text to solidify their claims and statements.

*This is a valid point and we have provided all the detailed information in the respective table. Since we always analysed between 30-36 individuals we state this initially when describing the procedure not to overload the ms with “n=>30” for 55 genes at different temperature and before and after developmental focusing in a total of more than 220 instances. We are confident that this is a good compromise giving the range initially (and all details in a table) without negatively impacting on the readability of the manuscript.*

7) Line 225 and other instances: cardiovascular diseases can be prevented if diagnosed early, but this mostly concerns acquired issues (blood pressure, clotting issues, etc.). The aeuthors' assay is more suited to uncover congenital issues affecting structure and overall function of the heart. Revisiting these statements throughout is recommended.

*We thank the referee for the comments here. While we agree that our assay uncovers congenital issues, the limitation does not reside within the assay or model system but is rather a reflection of the initial set of candidate genes, i. e. the human GWAS itself. We have incorporated that into the revised version of the manuscript.*

8) Line 244 ff is a prime example for how the authors omit prior work (unnecessarily so) by discussing prior zebrafish work without providing a single reference. Similarly line 254, while possibly true for Medaka, lots of prior refinements in using RNPs, salt-based solubilization, etc. has been done in other models.

*We thank the referee to point this our and apologize for our mistake. We have now incorporated the respective references.*

9) The authors argue that AI is required for deeper phenotyping of heart issues. The reviewer would argue that decades imaging and developmental biology work has shown pretty well that grad students, postdocs, and other lab personnel with sufficient training and expertise can manage to decode cardiac phenotypes. This should be rephrased or further explained, i.e. how AI would actually help (i.e. in combo with high-throughput imaging, etc.).

*The referee is absolutely correct in his statement and AI is not meant to replace experience. What we intended to say is that automated high throughput analyses addressing multiple features in parallel will profit from AI and rather frees the experts from repetitive work unleashing their creativity. Trained personnel can definitely do the job, but it could take as much as a whole day to reliably quantify complex morphological phenotypes for individual embryos in a 96 well plate thus significantly delaying data interpretation and target gene identification. Trained AI platforms reliably and automatically extracting said phenotypes in the background not only amplify phenotypic data acquisition but also allow the highly trained personnel to invest their time and effort in doing other experiments.*

*We have rephrased the paragraph to clarify our point.*
